# The Treatment and Outcome of Patients With Soft Tissue Sarcomas and Synchronous Metastases

**DOI:** 10.1080/1357714021000022168

**Published:** 2002-06

**Authors:** John M. Kane, J. William Finley, Deborah Driscoll, William G. Kraybill, John F. Gibbs

**Affiliations:** Division of Surgical Oncology Roswell Park Cancer Institute Elm & Carlton Streets Buffalo NY 14263 USA

## Abstract

*Introduction:* There is a strong association between poor overall survival and a short disease-free interval for patients with soft tissue sarcomas (STS) and metastatic disease. Patients with STS and synchronous metastases should have a very dismal prognosis.The role of surgery in this subgroup of patients with STS has not been defined.

*Patients and Methods:* A single-institution retrospective review was performed of 48 patients with STS and synchronous metastases in regard to patient demographics, presentation, tumor characteristics, metastatic sites, treatment, follow-up, and survival over a 27-year period.

*Results:* Most primary tumors were ≥10 cm (58%), high-grade histology (77%), and located on the extremity (60%).The most frequent site of metastatic disease was the lung (63%); 27% of patients had metastases to ≥2 organ sites. Surgery to the primary tumor was performed in 94% of patients (*n* = 45) and 68% had additional radiation therapy (*n* = 32). Thirty- five percent of patients underwent at least one metastastectomy (*n* = 17). Chemotherapy was administered to 90% of patients (*n* = 43); 31% received ≥3 different regimens (*n* = 15) and 25% were given intra-arterial or intracavitary therapy (*n* = 12). Median overall survival was 15 months with a 21% 2-year survival. Local control of the primary tumor was achieved in 54% (*n* = 26), and metastastectomy was performed in 35% (*n* = 17). No analyzed factors were associated with an improvement in overall survival

*Conclusions:* Despite multiple poor prognostic factors, the survival of patients with STS and metastases is comparable to those who develop delayed metastatic disease. However, unlike patients who present with metachronous disease, there was no improved survival observed for patients treated with metastastectomy. Consequently, treatment for patients with STS and synchronous metastases should be approached with caution. Surgical management of STS with synchronous metastases must be considered palliative and should be reserved for patients requiring palliation of symptoms. Patients must also be well informed of the noncurative nature of the procedure.


*Sarcoma* (2002) **6**, 69–73

Taylor&Francis healthsciences

**ORIGINAL ARTICLE**

### The treatment and outcome of patients with soft tissue sarcomas and synchronous metastases

JOHN M. KANE III, J. WILLIAM FINLEY, DEBORAH DRISCOLL, WILLIAM G. KRAYBILL & JOHN F. GIBBS

*Division of Surgical Oncology, Roswell Park Cancer Institute, Elm & Carlton Streets, Buffalo, NY 14263, USA*

**Abstract**

*Introduction*: There is a strong association between poor overall survival and a short disease-free interval for patients with soft tissue sarcomas (STS) and metastatic disease. Patients with STS and synchronous metastases should have a very dismal prognosis. The role of surgery in this subgroup of patients with STS has not been defined.

*Patients and Methods*: A single-institution retrospective review was performed of 48 patients with STS and synchronous metastases in regard to patient demographics, presentation, tumor characteristics, metastatic sites, treatment, follow-up, and survival over a 27-year period.

*Results*: Most primary tumors were ≥10 cm (58%), high-grade histology (77%), and located on the extremity (60%). The most frequent site of metastatic disease was the lung (63%); 27% of patients had metastases to ≥ 2 organ sites. Surgery to the primary tumor was performed in 94% of patients ($n$ = 45) and 68% had additional radiation therapy ($n$ = 32). Thirty-five percent of patients underwent at least one metastastectomy ($n$ = 17). Chemotherapy was administered to 90% of patients ($n$ = 43); 31% received ≥3 different regimens ($n$ = 15) and 25% were given intra-arterial or intracavitary therapy ($n$ = 12). Median overall survival was 15 months with a 21% 2-year survival. Local control of the primary tumor was achieved in 54% ($n$ = 26), and metastastectomy was performed in 35% ($n$ = 17). No analyzed factors were associated with an improvement in overall survival.

*Conclusions*: Despite multiple poor prognostic factors, the survival of patients with STS and metastases is comparable to those who develop delayed metastatic disease. However, unlike patients who present with metachronous disease, there was no improved survival observed for patients treated with metastastectomy. Consequently, treatment for patients with STS and synchronous metastases should be approached with caution. Surgical management of STS with synchronous metastases must be considered palliative and should be reserved for patients requiring palliation of symptoms. Patients must also be well informed of the noncurative nature of the procedure.

**Key words**: *sarcoma, synchronous metastases, metachronous metastases, outcome, surgery*

### Introduction

Soft tissue sarcomas (STS) are a relatively rare, heterogeneous group of tumors that arise from mesenchymal tissue. It is estimated 8300 new cases of STS were diagnosed in the United States in 2002.[1] The treatment of STS has been primarily surgical, with adjuvant therapy based on the size of the tumor, grade, and margin status. Using a multimodality approach, local recurrence rates are less than 20% of patients with STS of the extremity.[2] Adequate control of intra-abdominal and retroperitoneal tumors has been more problematic. Despite significant improvements in the rates of local control and limb salvage, it is the development of distant metastases that remains the major determinant of mortality from STS.

Approximately 20–38% of patients with STS will eventually develop metastatic disease.[3–6] The lungs are the predominant sites of distant disease. Although less common, metastases to lymph nodes, the liver, the peritoneum, soft tissue, bone, and the central nervous system also occur. The development of regional and/or distant metastases is associated with extremely poor survival.

Several factors have been associated with the survival of sarcoma patients with metastatic disease. These include age, surgical treatment of the metastases, local recurrence, and tumor size.[4,7] A

Correspondence to: John F. Gibbs, M.D., Division of Surgical Oncology, Roswell Park Cancer Institute, Elm & Carlton Streets, Buffalo, NY 14263, USA. Tel.: +1–716–845–5807; Fax: +1–716–845–3434.

ISSN 1357–714X print/ISSN 1369–1643 online/02/0200069–05 © Taylor & Francis Ltd
DOI: 10.1080/1357714021000022168

prolonged disease-free interval from the time of diagnosis until the development of distant metastases has also been linked to an improved survival.[4,8,9] Based on this observation, patients with STS and synchronous metastases should have a dismal prognosis.

A difficult dilemma surrounds the management of this group of patients with STS who present with synchronous metastases. Although an improved survival has been demonstrated in patients with surgically resected metachronous metastases with STS,[10,11] similar information is not available for patients with STS and synchronous metastases. The purpose of this study was to describe the experience at a tertiary care cancer center, identify prognostic factors associated with improved survival, and to assess the impact of surgical intervention on their outcome.

### Patients and methods

A retrospective review of the Roswell Park Cancer Institute (RPCI) Tumor Registry from 1971 to 1998 was performed to identify patients with the diagnosis of a soft tissue sarcoma and synchronous metastases. Sarcomas felt to be of bone origin were excluded from the review. Histological diagnosis of soft tissue sarcoma was performed or confirmed by RPCI pathologists. Fifty-three patients were identified as having a soft tissue sarcoma with synchronous metastases. Review of the medical records revealed five patients who did not meet the definition of synchronous metastases. Four patients had delayed development of metastatic disease and one patient had a synchronous second non-sarcomatous primary tumor. Consequently, the study group for this study consisted of 48 patients. The diagnosis of metastatic disease was established by biopsy of suspicious lesions or strongly suspected based on radiological studies and confirmed by subsequent progression of disease. For the purpose of this study, synchronous was defined as either: (1) metastatic disease present at the time of pathological diagnosis of the primary tumor, or (2) metastatic disease present at the time of the first surgical intervention for the primary tumor.

A complete review of the RPCI medical record and available outside information was performed for all patients identified from the Tumor Registry. Information obtained included: patient demographics (age, gender, race), presentation (symptoms, diagnosis, work-up), tumor characteristics (histology, size, location, metastatic sites), treatment (surgery, chemotherapy, radiation), follow-up, and survival. The study was approved by the Roswell Park Cancer Institute Institutional Review Board. Patient confidentiality was maintained.

Estimated overall survival was calculated by the method of Kaplan and Meier. Tests of significance with respect to survival distributions were based on the log-rank test. Statistical significance for all analyses was defined as $p < 0.05$. All statistical calculations were performed using SPSS for Windows (ver. 6.1).

### Results

Demographic information and tumor characteristics for the 48 patients are summarized in Table 1. This study population includes a wide variety of histological subtypes.

The diagnosis of metastases was made pre-operatively in 40 patients (83%) and at the time of the first surgery in eight patients (17%). Seventy-three percent of patients ($n$ = 35) had metastases limited to one anatomic site at the time of diagnosis (Table 2). Synchronous pulmonary metastases were most commonly encountered ($n$ = 30). Nodal disease was found in 11 patients. Hepatic involvement was a rare event ($n$ = 4).

Surgery played an integral role at our institution in the treatment of both the primary tumor and the metastatic disease. Forty-five patients underwent at least one attempt at surgical resection of the primary tumor and two or more operations were performed on 13 patients. Seven patients had an unresectable primary tumor at the time of surgical intervention, three of which had concomitant sarcomatosis. Seventeen patients underwent at least one attempt at metastasectomy. Resection of pulmonary metastases was the most common procedure ($n$ = 13) with four patients undergoing two or more operations (Table 3). The median survival of patients who underwent at least one metastasectomy procedure was 16.0 versus 14 months for those with no attempt at resection of metastatic disease (Fig. 1, $p$ = 0.3).

Chemotherapy was given to 43 of 48 patients (90%), with 15 patients (31%) receiving three or more different chemotherapeutic regimens. The majority also received at least one regimen of doxorubicin based therapy ($n$ = 40, 93%). Seven patients were treated with chemotherapy prior to any attempt at surgical resection of the primary tumor. Twenty-eight percent of patients receiving chemotherapy ($n$ = 12) were given at least one regimen through a non-intravenous route. Intra-arterial doxorubicin was administered into the extremity in seven patients, via the hepatic artery in three patients, and via the pulmonary artery in two patients. Cisplatin was given intraperitoneally to two patients and intrathoracically to one patient. Sixteen patients received radiation therapy to the primary tumor site.

The overall median survival was 15.0 months. At the time of last follow-up, 88% of the patients had died ($n$ = 42). Three patients are alive with disease at 15, 9, and 6 months. An additional three patients are alive with no evidence of disease at 19, 8, and 8 months. Overall survival is presented in Fig. 2. The effect of various factors on overall survival is shown in Table 4. None of the examined variables had a statistically significant impact on overall survival.

**Table 1.** *Characteristics of 48 patients with a soft tissue sarcoma and synchronous metastases*

| | | |
|---|---|---|
| *Age (years)* | | |
| Mean | 44.8 | |
| Range | 13–83 | |
| < 50 | 52% | (25) |
| > 50 | 48% | (23) |
| *Sex* | | |
| Male | 60% | (29) |
| Female | 40% | (19) |
| *Race* | | |
| Caucasian | 88% | (42) |
| African-American | 8% | (4) |
| Hispanic | 4% | (2) |
| *Interval form symptoms to diagnosis (months)* | | |
| Mean | 6.7 | |
| Median | 3.1 | |
| Range | 0–36 | |
| *Symptoms* | | |
| Mass | 67% | (32) |
| Pain | 60% | (29) |
| Weight loss | 8% | (4) |
| Fevers | 4% | (2) |
| *Size (cm)* | | *n*=45 |
| Mean | 11 | |
| Range | 2.5–40 | |
| < 10 | 42% | (19) |
| > 10 | 58% | (26) |
| *Grade* | | |
| High | 77% | (37) |
| Intermediate | 8% | (4) |
| Unspecified | 15% | (7) |
| *Site* | | |
| Extremity | 60% | (29) |
| Intra-abdominal | 27% | (13) |
| Trunk | 13% | (6) |
| *Histology* | | |
| Leiomyosarcoma | 21% | (10) |
| Gastrointestinal stromal tumor (GIST) | 15% | (7) |
| Malignant fibrous histiocytoma | 10% | (5) |
| Synovial cell | 10% | (5) |
| Liposarcoma | 6% | (3) |
| Undifferentiated | 6% | (3) |
| Other | 31% | (15) |

**Table 2.** *Characteristics of the metastatic tumor in 48 patients with a soft tissue sarcoma and synchronous metastases*

| | | |
|---|---|---|
| *Site* | | |
| Lung | 63% | (30) |
| Lymph nodes | 23% | (11) |
| Peritoneal | 19% | (9) |
| Soft tissue/bone | 17% | (8) |
| Liver | 8% | (4) |
| Bone marrow | 2% | (1) |
| *Number of sites* | | |
| One | 73% | (35) |
| Two | 23% | (11) |
| Three | 4% | (2) |

**Table 3.** *Surgical treatment of metastatic tumor sites in 48 patients with soft tissue sarcomas and synchronous metastases*

| | | |
|---|---|---|
| *Any metastasectomy* | | |
| No | 65% | (31) |
| Yes | 35% | (17) |
| *Number of pulmonary metastasectomies* | | |
| None | 73% | (35) |
| One | 19% | (9) |
| ≥ Two | 8% | (4) |
| Range | 0–4 | |

**Table 4.** *Effect of various factors on the overall survival in 48 patients with soft tissue sarcomas and synchronous metastases (statistically significant at $p < 0.05$)*

| Variable | *p* value |
|---|---|
| Sex | 0.23 |
| Age (< vs. ≥ 50 years) | 0.53 |
| Primary tumor histology | 0.51 |
| Grade | 0.28 |
| Size (< vs. ≥ 10 cm) | 0.74 |
| Primary tumor site | 0.93 |
| Number of surgeries at primary site | 0.68 |
| Residual tumor after primary surgery | 0.93 |
| Metastasectomy | 0.30 |
| Chemotherapy cycles (< vs. ≥ 3) | 0.22 |
| Pre-resection chemotherapy | 0.81 |
| Non-intravenous chemotherapy | 0.58 |
| Radiation to primary tumor site | 0.69 |
| Control of primary tumor site | 0.60 |

### Discussion

The development of metastases from STS carries a poor prognosis. With metastatic disease, 62–80% of patients will have pulmonary metastases and 50–70% will have isolated pulmonary metastases.[4,8,10,12,13] Lymph node metastases from STS are generally considered a rare event, present in only 2.6–16% of all patients with STS.[14,15] Approximately 1.2–6.6% of all patients with STS develop soft tissue metastases, usually as a late event associated with widely disseminated disease.[16] Median survival following the diagnosis of metastatic disease ranges from 8 to 14.5 months.[3,4,7,12] Two-year overall survival is 20–28%, decreasing to 10% for patients with multiple sites of metastatic disease.[3,4,12] Several prognostic factors for overall survival of patients with metastatic STS have been identified. Tumor size >10 cm, local recurrence, unresectable metastases, age >50 years, and a disease-free interval <1 year have all been associated with a decreased survival after the development of metastases.[4,7,8]

The proportion of patients with STS who present with synchronous metastatic disease ranges from 12 to 23% in uncontrolled series and represents a subgroup of patients with no disease-free interval.[5,6] Suit

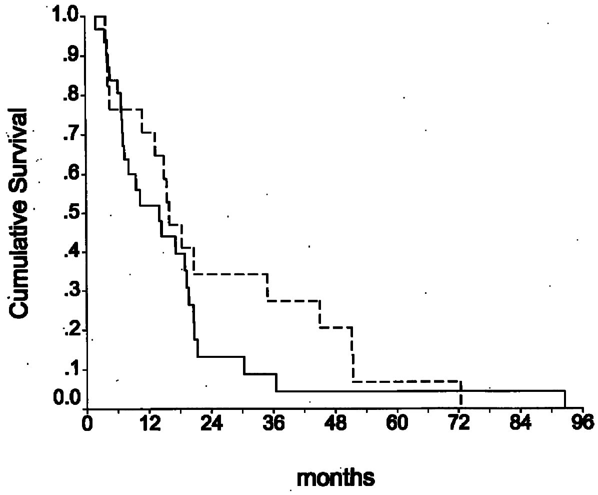

**Fig. 1.** *Survival by surgery for metastatic disease. Kaplan–Meier survival curves are presented with reference to surgical intervention for STS with synchonous metastases. Survival for patients who underwent metastastectomy is represented by the solid line (*n *= 31; 26 failures; median survival 1 4 months). Survival for patients who did not undergo metastastectomy is represented by the dashed line (*n *= 17; 16 failures; median survival 16 months). There was no difference in survival (*p *= 0.3).*

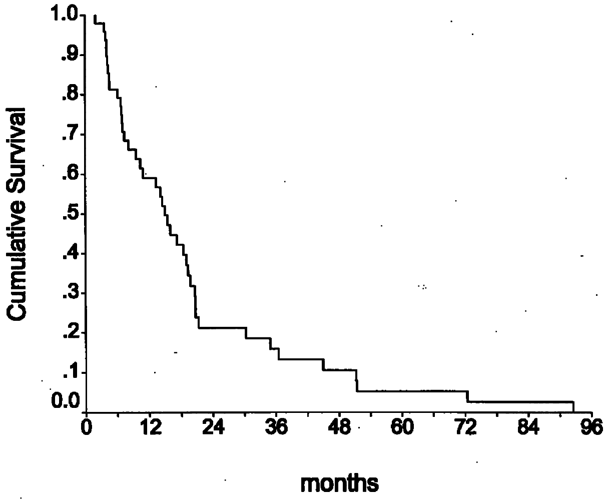

**Fig. 2.** *Overall survival. A Kaplan–Meier curve is presented for overall survival in for patients presenting with STS and synchronous metastases (*n *= 48; 42 failures; median survival 15 months). Overall 2-year survival was 21%.*

reported 1.6% of patients with tumors <5 cm had synchronous metastases as opposed to 13% for tumors >5cm.[5] In the subgroup of patients with poorly differentiated tumors, >5cm, 20% had metastatic disease at presentation. Those patients who present with synchronous metastases represent a difficult subgroup to manage. Our approach to this type of patient has been traditionally aggressive surgery for the primary and metastatic sites. This approach allows us to fully evaluate the role of surgery in these patients. The median overall survival in this series was 15.0 months with 21% of patients alive at 2 years. Ninety-four percent of patients had surgery at the site of the primary tumor and local control was eventually achieved in over one-half of all patients. Thirty-five percent of patients underwent at least one attempted metastastectomy; 76% of these were for pulmonary metastases. Although the median survival for patients undergoing metastastectomy was slightly longer that of the patients deemed unresectable, the difference was not statistically significant. The benefit of pulmonary metastastectomy for STS in selected patients with metachronous metastases has a 3-year overall survival of 46–54%.[10,11] Prognostic factors identified for extended survival following pulmonary metastastectomy include an extended disease-free interval and complete metastasectomy.[10,11,17]

We did not observe an improved overall survival in those patients who underwent metastastectomy versus those deemed unresectable. Several factors may present difficulties in the interpretation of the data. The patient population included in this series was comprised of a small sample size identified over a 27-year period. During that period of time, medical technology has evolved. For example, computer tomography most assuredly permitted earlier diagnosis of pulmonary metastatic disease in 1998 as compared with 1971 when only chest X-ray was available. Additionally, analysis of several factors failed to identify any variable associated with improved overall survival. The uniform outcome of the patients in our study is most likely due to the fact that all patients had similar synchronous metastatic tumor burdens that nullified the predictive value of standard prognostic factors.

Given the disseminated nature of their disease, the majority of patients received systemic chemotherapy (90%), usually consisting of at least one doxorubicin-based regimen. The overall benefit of chemotherapy in the treatment of metastatic STS is somewhat controversial. Response rates have ranged from 15 to 48%, but there has been little impact on overall survival.[2,18,19] Approximately one-third of the patients was treated with three or more different chemotherapy regimens with no apparent impact on survival. It is conceivable the various chemotherapeutic regimens or radiation therapy had an impact on survival. However, given the diversity of regimens, the heterogeneous tumor histology present, variations in biological response to non-surgical therapy, and the small sample size, it is impossible to determine the presence of any effect.

Despite the fact that there is no disease-free interval, the survival of patients with STS and synchronous metastases is comparable to those who develop delayed metastatic disease. However, there does not appear to be any added survival benefit of metastastectomy for patients with STS and synchronous metastases as has been observed in metastastectomy for metachronous metastases. Although this is a small retrospective series of patients over a large period of time, the results appear to argue against the surgical management for the purpose of extending survival for patients with STS and synchronous metastases. Clearly, there remains a role for surgery in this group of patients when there is palliative intent for the surgery, i.e., maintenance and/or improvement in the quality of life. Nevertheless, the surgical management of the patient who presents with STS and synchronous metastatic disease, must be approached with caution, the goals, and risks of morbidity and mortality of surgery clearly delineated, and patient expectations thoroughly discussed. Further study is warranted regarding the role of multimodality therapy in this difficult dilemma.

### References

1. Jemal A, Thomas A, Murray T, Thun M. Cancer Statistics, 2002. *CA Cancer J Clin* 2002; 52: 23–47.
2. Valle AA, Kraybill WG. Management of soft tissue sarcomas of the extremity in adults. *J Surg Oncol* 1996; 63: 271–9.
3. Brennan MF, Casper ES, Harrison LB. Soft tissue sarcoma. In: DeVita VT, Hellman S, Rosenberg SA, eds. *Cancer Principles and Practice of Oncology*. Philadelphia, PA: Lippincott-Raven, 1997: 1738–88.
4. Billingsley KG, Lewis JJ, Leung DH, *et al.* Multifactorial analysis of the survival of patients with distant metastases arising from primary extremity sarcoma. *Cancer* 1999; 85: 389–95.
5. Suit HD. Patterns of failure after treatment of sarcoma of soft tissue by radical surgery or by conservative surgery and radiation. *Cancer Treat Symp* 1983; 2: 241–6.
6. Lawrence W, Donegan WL, Natarajan N, *et al.* Adult soft tissue sarcomas: a pattern of care survey of the American College of Surgeons. *Ann Surg* 1987; 205: 349–59.
7. Pisters PWT, Leung DHY, Woodruff J, *et al.* Analysis of prognostic factors in 1,041 patients with localized soft tissue sarcomas of the extremities. *J Clin Oncol* 1996; 14: 1679–89.
8. Torosian MH, Friedrich C, Godbold J, *et al.* Soft tissue sarcoma: initial characteristics and prognostic factors in patients with and without metastatic disease. *Semin Surg Oncology* 1988; 4: 13–9.
9. Gibbs JF, Lee RJ, Driscoll DL, McGrath BE, Mindell ER, Kraybill WG. Clinical importance of late recurrence in soft tissue sarcoma. *J Surg Oncol* 2000; 73:81–86.
10. Van Geel AN, Pastorino U, Jauch KW, *et al.* Surgical treatment of lung metastases: the European Organization for Research and Treatment of Cancer-Soft Tissue and Bone Sarcoma Group study of 255 patients. *Cancer* 1996; 77: 675–82.
11. Billingsley KG, Burt ME, Jara E, *et al.* Pulmonary metastases from soft tissue sarcoma: analysis of patterns of disease and postmetastasis survival. *Ann Surg* 1999; 229: 602–12.
12. Huth JF, Eilber FR. Patterns of metastatic spread following resection of extremity soft tissue sarcomas and strategies for treatment. *Semin Surg Oncol* 1988; 4: 20–6.
13. Vezeridis MP, Moore R, Karakousis CP. Metastatic patterns in soft tissue sarcomas. *Arch Surg* 1983; 118; 915–8.
14. Fong Y, Coit DG, Woodruff JM, *et al.* Lymph node metastases from soft tissue sarcomas in adults. *Ann Surg* 1993; 217: 72–7.
15. Ariel IM. Incidence of metastases to lymph nodes from soft tissue sarcomas. *Sem Surg Oncol* 1988; 4: 27–9.
16. Rao UNM, Hanan SH, Lotze MT, *et al.* Distant skin and soft tissue metastases from sarcomas. *J Surg Oncol* 1998; 69: 94–8.
17. Putnam JB. Soft part sarcomas-metastases. *Chest Surg Clin N Am* 1998; 8: 97–118.
18. Bramwell VHC. Chemotherapy for metastatic soft tissue sarcomas-another full circle? *Br J Cancer* 1991; 64: 7–9.
19. Sawyer M, Bramwell V. The treatment of distant metastases in soft tissue sarcoma. *Semin Radiat Oncol* 1999; 9: 389–400.

## References

[PDF_00464] Ariel I. M. (1988). Incidence of metastases to lymph nodes from soft-tissue sarcomas.. Semin Surg Oncol.

[PDF_00450] Billingsley K. G., Burt M. E., Jara E., Ginsberg R. J., Woodruff J. M., Leung D. H., Brennan M. F. (1999). Pulmonary metastases from soft tissue sarcoma: analysis of patterns of diseases and postmetastasis survival.. Ann Surg.

[PDF_00421] Billingsley K. G., Lewis J. J., Leung D. H., Casper E. S., Woodruff J. M., Brennan M. F. (1999). Multifactorial analysis of the survival of patients with distant metastasis arising from primary extremity sarcoma.. Cancer.

[PDF_00472] Bramwell V. H. (1991). Chemotherapy for metastatic soft tissue sarcomas--another full circle?. Br J Cancer.

[PDF_00461] Fong Y., Coit D. G., Woodruff J. M., Brennan M. F. (1993). Lymph node metastasis from soft tissue sarcoma in adults. Analysis of data from a prospective database of 1772 sarcoma patients.. Ann Surg.

[PDF_00441] Gibbs J. F., Lee R. J., Driscoll D. L., McGrath B. E., Mindell E. R., Kraybill W. G. (2000). Clinical importance of late recurrence in soft-tissue sarcomas.. J Surg Oncol.

[PDF_00454] Huth J. F., Eilber F. R. (1988). Patterns of metastatic spread following resection of extremity soft-tissue sarcomas and strategies for treatment.. Semin Surg Oncol.

[PDF_00412] Jemal Ahmedin, Thomas Andrea, Murray Taylor, Thun Michael (2002). Cancer statistics, 2002.. CA Cancer J Clin.

[PDF_00429] Lawrence W., Donegan W. L., Natarajan N., Mettlin C., Beart R., Winchester D. (1987). Adult soft tissue sarcomas. A pattern of care survey of the American College of Surgeons.. Ann Surg.

[PDF_00433] Pisters P. W., Leung D. H., Woodruff J., Shi W., Brennan M. F. (1996). Analysis of prognostic factors in 1,041 patients with localized soft tissue sarcomas of the extremities.. J Clin Oncol.

[PDF_00470] Putnam J. B. (1998). Soft part sarcomas--metastases.. Chest Surg Clin N Am.

[PDF_00467] Rao U. N., Hanan S. H., Lotze M. T., Karakousis C. P. (1998). Distant skin and soft tissue metastases from sarcomas.. J Surg Oncol.

[PDF_00475] Sawyer M., Bramwell V. (1999). The treatment of distant metastases in soft tissue sarcoma.. Semin Radiat Oncol.

[PDF_00437] Torosian M. H., Friedrich C., Godbold J., Hajdu S. I., Brennan M. F. (1988). Soft-tissue sarcoma: initial characteristics and prognostic factors in patients with and without metastatic disease.. Semin Surg Oncol.

[PDF_00414] Valle A. A., Kraybill W. G. (1996). Management of soft tissue sarcomas of the extremity in adults.. J Surg Oncol.

[PDF_00458] Vezeridis M. P., Moore R., Karakousis C. P. (1983). Metastatic patterns in soft-tissue sarcomas.. Arch Surg.

[PDF_00445] van Geel A. N., Pastorino U., Jauch K. W., Judson I. R., van Coevorden F., Buesa J. M., Nielsen O. S., Boudinet A., Tursz T., Schmitz P. I. (1996). Surgical treatment of lung metastases: The European Organization for Research and Treatment of Cancer-Soft Tissue and Bone Sarcoma Group study of 255 patients.. Cancer.

